# Dystocia in cattle and horses: a compilation of historical artworks dedicated to Professor Gerhard Sand (1861–1921)

**DOI:** 10.1186/s13028-024-00733-1

**Published:** 2024-03-15

**Authors:** Jørgen Steen Agerholm, Mette Christoffersen, Jan Boysen-Møller Secher, Annika Normann, Hanne Gervi Pedersen

**Affiliations:** 1 Section for Veterinary Reproduction and Obstetrics, Department of Veterinary Clinical Sciences, University of Copenhagen, Højbakkegaard Allé 5A, DK-2630 Taastrup, Denmark; 2 Department of Veterinary and Animal Sciences, University of Copenhagen, Grønnegårdsvej 15, DK-1870 Frederiksberg C, Denmark

**Keywords:** Calving, Foaling, Obstetrics, Sand emasculator castration clamp, *Streptococcus equi*, Teaching, Veterinary curriculum

## Abstract

Professor Gerhard Sand (1861–1921) was the first professor of veterinary obstetrics at the Royal Veterinary and Agricultural University, Copenhagen, Denmark. He began teaching the theory and practice of obstetrics to veterinary students in 1887 and spent the following years until his death in 1921 developing the veterinary obstetrics teaching program. During this period, veterinary obstetrics was established as an independent discipline at the Royal Veterinary and Agricultural University. Professor Sand’s teaching had a major impact on the field of veterinary obstetrics in Scandinavia. He was devoted to teaching veterinary obstetrics and produced a number of obstetrical illustrations, some of which showed different causes of dystocia, mainly fetal malpresentation in cattle and horses. Professor Sand created the illustrations with the intention of publishing a handbook of obstetrics, but due to illness and an early death, this work was never completed. This compilation of historical artworks of dystocia in cattle and horses includes some of these illustrations and is published to honour Professor Sand, with the intention of making his illustrations widely available for the teaching of veterinary obstetrics.

**Supplementary Information:**

The online version contains supplementary material available at 10.1186/s13028-024-00733-1.

## Background

**Fig. 1:**
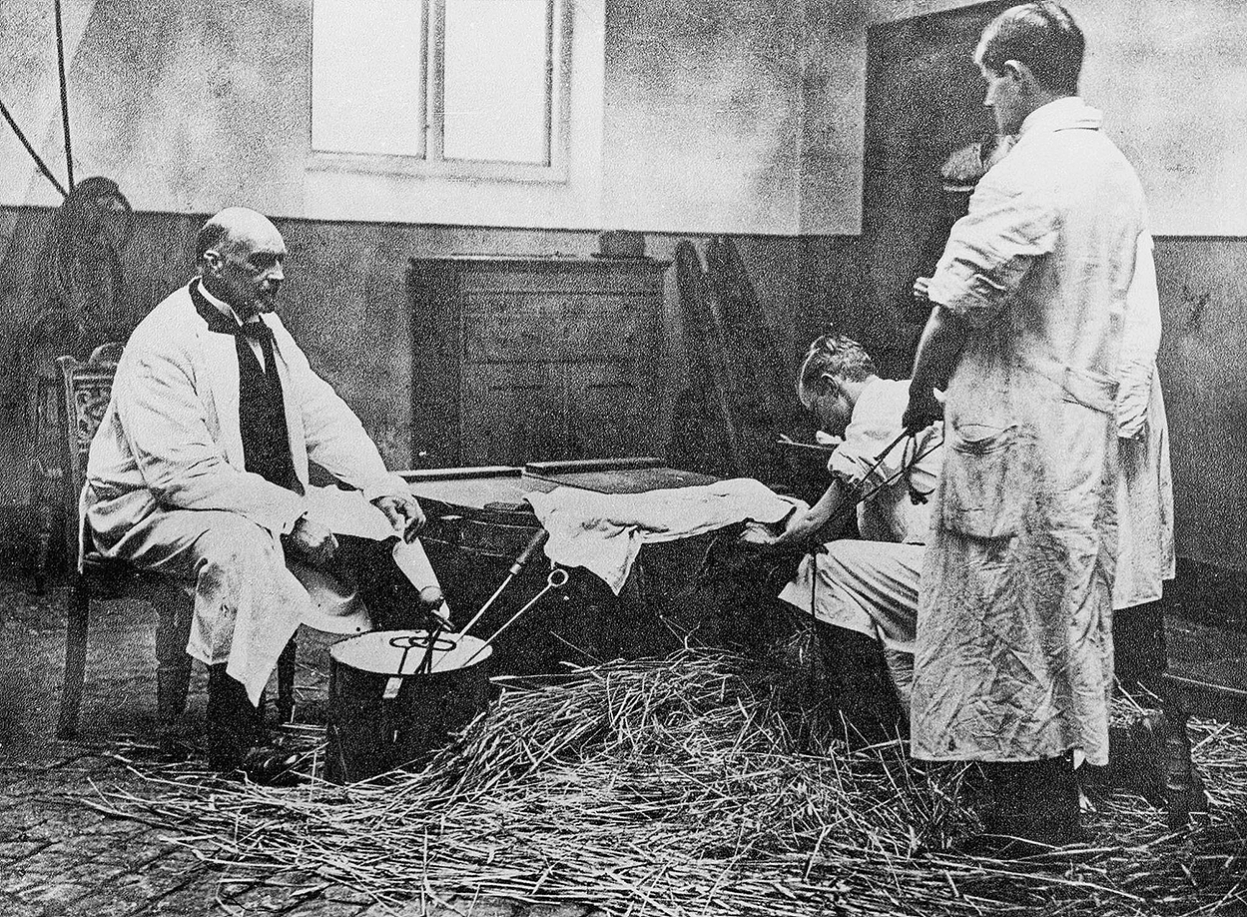
Professor Sand (sitting to the left) supervising a training session for veterinary students in handling dystocia and performing fetotomy in a wooden box– an early model of a dystocia simulator. Photo taken ca. 1910–1920

Dystocia is a severe complication of parturition that requires immediate intervention by trained individuals to minimize the associated pain, avoid birth trauma and ensure the survival and health of the fetus/fetuses and the dam. Handling of parturient animals and professional intervention in case of complications (veterinary obstetrics) are therefore important skills for veterinarians that must be taught during their veterinary education. The significance of veterinary obstetrics in Denmark is emphasized in the law as the Danish Act on Veterinarians states that a veterinary practitioner is obliged to give the necessary assistance to animals with dystocia if called upon [1]. Training in obstetrics has therefore been a formal part of the veterinary curriculum in Denmark for more than 100 years. It was established as a specific discipline in the veterinary curriculum in 1887 and Professor Gerhard Alexis Cappelen Sand (Fig. 1) became the first professor in veterinary obstetrics in Denmark [2].

Professor Sand was born on 7th February 1861 in the town of Lemvig in northern Denmark. He graduated with distinction from the Royal Veterinary and Agricultural University in Copenhagen, Denmark in April 1883. He was employed at the school’s stationary clinic shortly thereafter, where he remained until 1887, when he became head of the ambulatory clinic. He kept this position for the remainder of his career [3, 4].

Professor Sand had an interest in bacteriology, and in addition to his work at the stationary clinic, he investigated the bacteriological cause of a number of diseases, initially in collaboration with Professor Carl Julius Salomonsen (1847–1924) and later in collaboration with Professor Carl Oluf Jensen (1864–1934), the founder of the Danish Veterinary Institute (“Statens Veterinære Serumlaboratorium”). His collaboration with Professor Jensen led to the discovery that equine strangles is caused by *Streptococcus equi.* Together they performed a range of experimental inoculations in horses with a *Streptococcus* species isolated from cases of strangles and were able to reproduce the disease and re-isolate the bacterium. They also showed that the characteristics of the bacterium differed from other known streptococcal species. They published their observations in 1888 [5].

As head of the ambulatory clinic, Professor Sand became responsible for teaching veterinary surgery. During this period, he developed several instruments, including an emasculator castration clamp for use in stallions and steers; an instrument that is still used globally under the name “Sand’s emasculator castration clamp”.

**Fig. 2:**
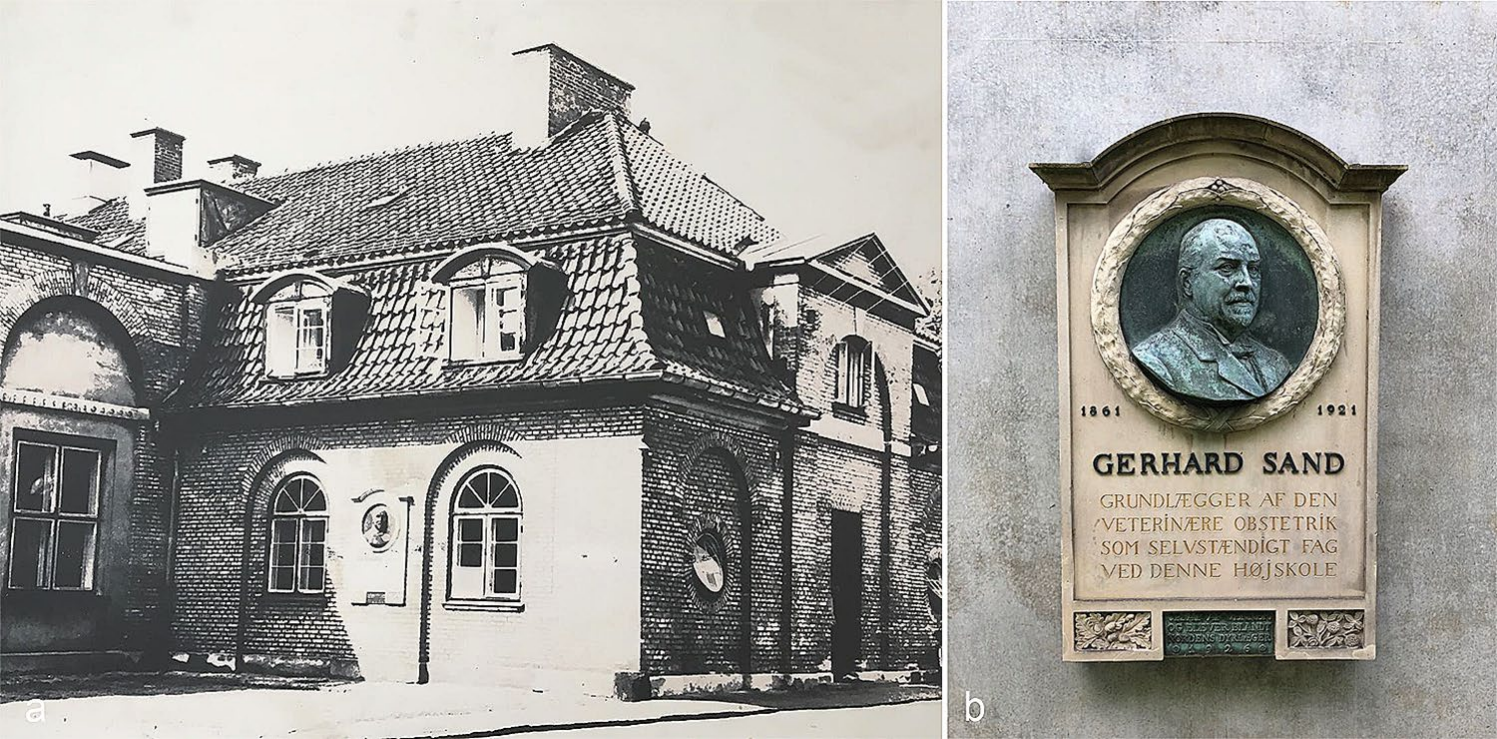
A commemorative plaque raised to honour Professor Sand for his contribution to the field of veterinary obstetrics. (**a**) The plaque was originally mounted on the (now demolished) obstetrics clinic on the Frederiksberg campus; (**b**) The plaque is now mounted on the building that houses the Section for Veterinary Reproduction and Obstetrics on the Taastrup campus. The inscription reads: “*Gerhard Sand. Founder of veterinary obstetrics as an independent subject at this college. Raised by Nordic veterinarian friends and students*”

Shortly after his employment as head of the ambulatory clinic, he began teaching the theory and practice of obstetrics to veterinary students. During the following years and until his death in 1921, he developed the veterinary obstetrics teaching program, and it was under his leadership that veterinary obstetrics was established as an independent discipline at the Royal Veterinary and Agricultural University in Copenhagen. The University was at that time the only veterinary school in Denmark, educating veterinarians from Denmark, Norway and Iceland. Professor Sand’s teaching therefore had a major impact on the field of veterinary obstetrics in Scandinavia, and to show their gratitude for his teaching, a commemorative plaque was raised by previous veterinary students and friends from Denmark and Norway. The plaque was placed on the building that (at that time) housed the veterinary obstetrics clinic (Fig. 2).

### Handbook in obstetrics

**Fig. 3:**
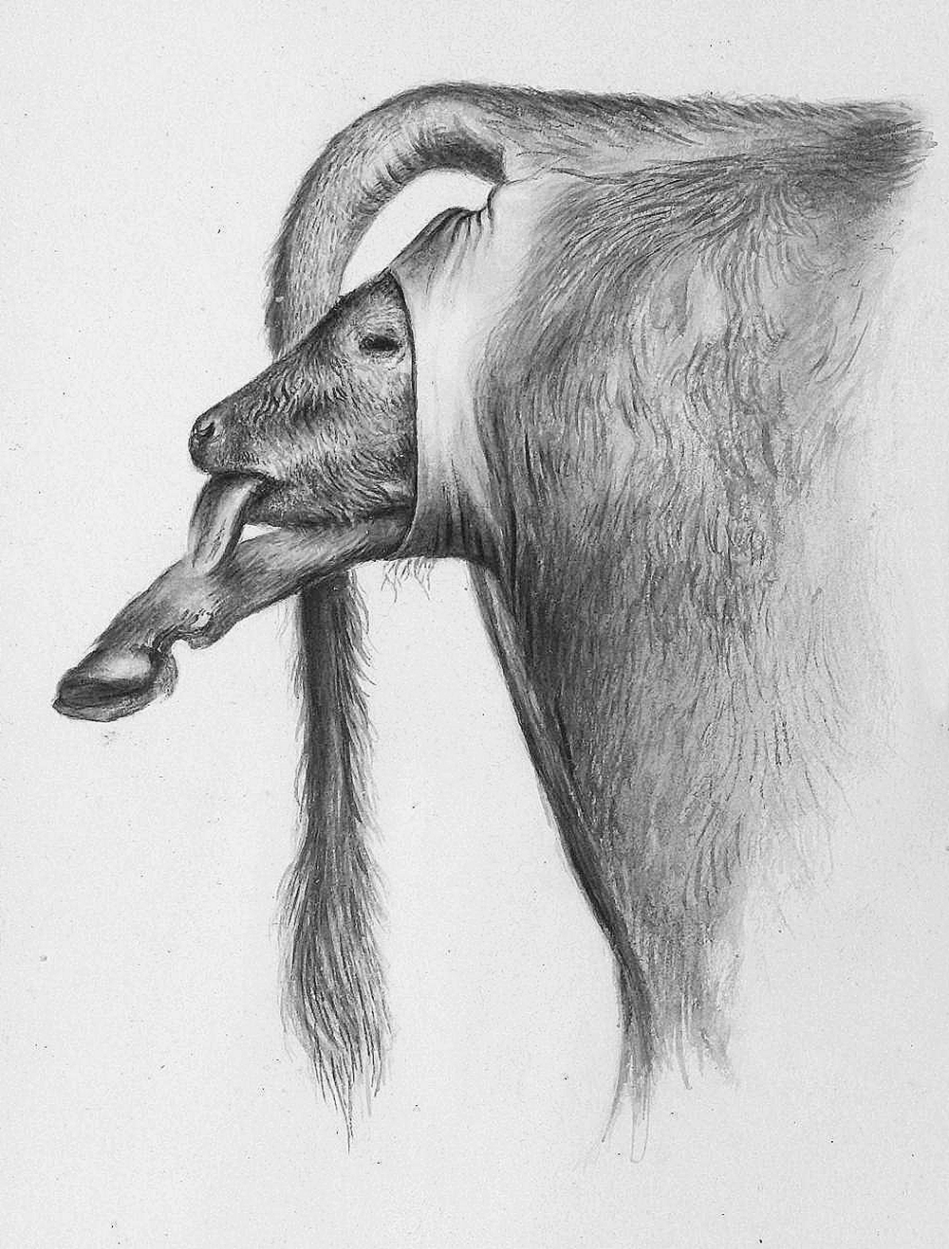
Bovine dystocia. Disposition of the calf: anterior presentation, dorsal position. Examination of the dam revealed a carpal flexion posture of the left forelimb. Drawing thought to be by CC Cordts from around the year 1900

During his career in veterinary obstetrics, Professor Sand produced a number of obstetrical illustrations, some of which showed causes of dystocia, mainly fetal malpresentation in cattle and horses. Some were based on intravenous formalin fixation of obstetrical cases and were made in collaboration with Simon Paulli (1865–1933), a professor of anatomy who had invented this technique [6, 7]. The full-body formalin fixation of obstetrical cases allowed in situ observation of the cause of dystocia and was at that time an excellent way to illustrate how dystocia can arise. The illustrations are unique, as full-body formalin fixation of bovine and equine fetuses is a technique rarely used today due to the toxicity of formaldehyde and the large volumes required. Professor Sand also collaborated with the illustrator Carl Christian Cordts (1844–1910), who produced a large number of zoological and anatomical drawings [8] in addition to illustrations showing obstetrical cases (Fig. 3).

Some of the original illustrations prepared by Professor Sand have been lost or are in poor condition, but illustrations of fetal malpresentation and cases of fetal malformation are available and in a suitable condition for reproduction. The most relevant illustrations have been included in this compilation of historical artworks of dystocia in cattle and horses.

Professor Sand created the illustrations with the intention of publishing a handbook in obstetrics, but due to illness and an early death, this work was never completed. However, some of the material was later included in a book on veterinary obstetrics written by Niels Ole Rasbech (1917–1988), professor of veterinary reproduction and obstetrics at the Royal Veterinary and Agricultural University, Copenhagen [9, 10].

### In honour of Professor Sand

Even today the illustrations based on the full body formalin-fixed obstetrical cases and drawings are of high educational value, as they allow for a better understanding of different types of fetal malpositioning. We are therefore publishing this compilation of historical artworks to ensure that these valuable illustrations are not lost for the future but are instead made available for teaching veterinary students worldwide. We believe that publishing Professor Sand’s illustrations in the open access journal *Acta Veterinaria Scandinavica* reflects his spirit, as he was highly dedicated to passing on knowledge and experience to veterinary students and to the veterinary community of the Nordic countries and worldwide.

We wish to honour Professor Sand for his valuable contribution to teaching veterinary obstetrics by publishing this compilation of historical artworks of dystocia in cattle and horses in connection with the 250th anniversary of the Danish veterinary education (2023).

### The illustrated atlas

**Fig. 4:**
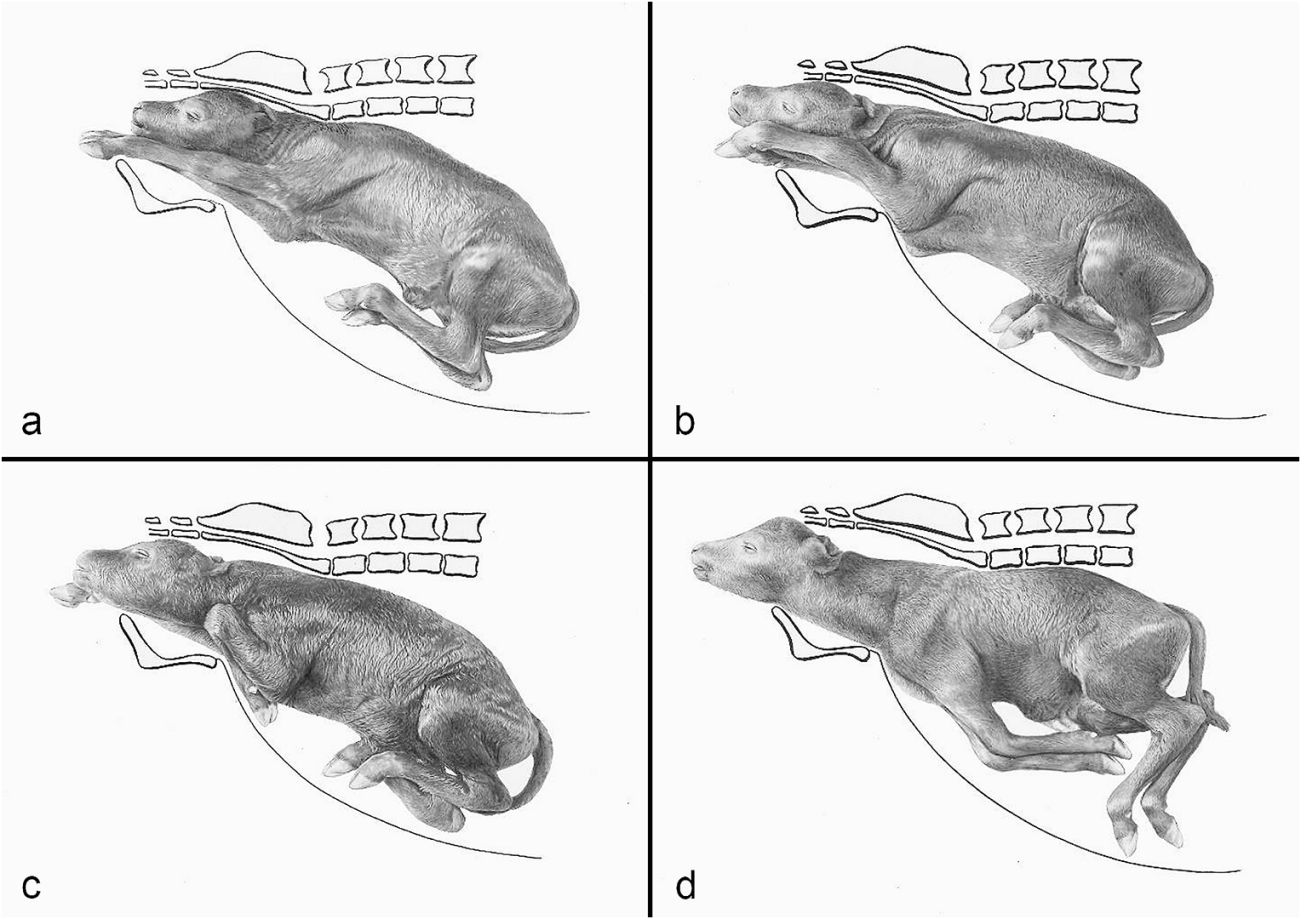
(**a**) Normal calving. Disposition of the calf: anterior presentation, dorsal position, normal posture; (**b–d**) Dystocias. (**b**) Anterior presentation, dorsal position, bilateral elbow flexion posture; (**c**) Anterior presentation, dorsal position, unilateral carpal flexion posture of the left forelimb; (**d**) Anterior presentation, dorsal position, bilateral shoulder flexion posture

**Fig. 5:**
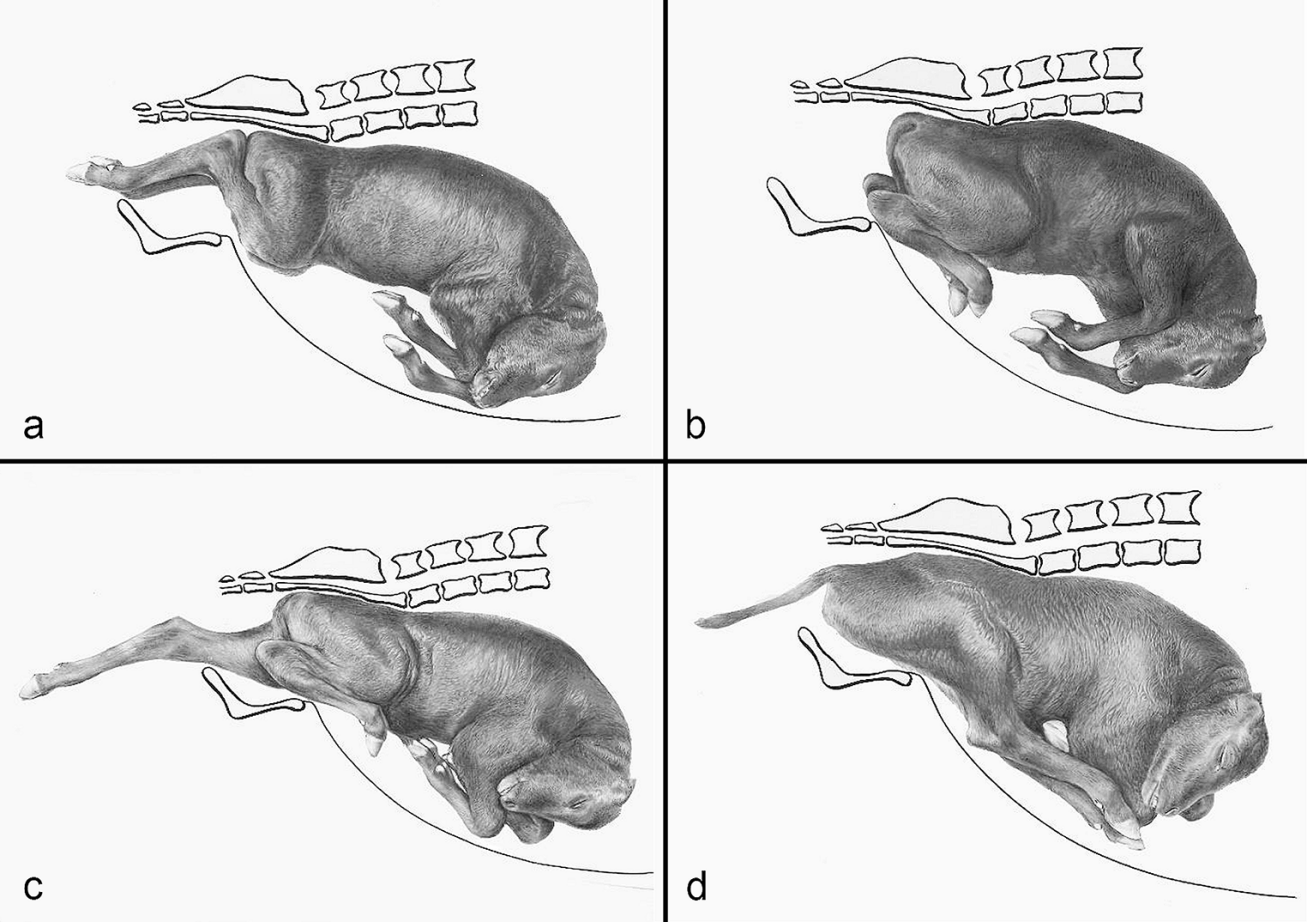
Bovine dystocia. Disposition of the calf: (**a**) Posterior presentation, dorsal position, bilateral stifle and hip flexion posture; (**b**) Posterior presentation, dorsal position, bilateral hock flexion posture; (**c**) Posterior presentation, dorsal position, unilateral hock flexion posture of the right hindlimb; (**d**) Posterior presentation, dorsal position, bilateral hip flexion posture (breech presentation)

**Fig. 6:**
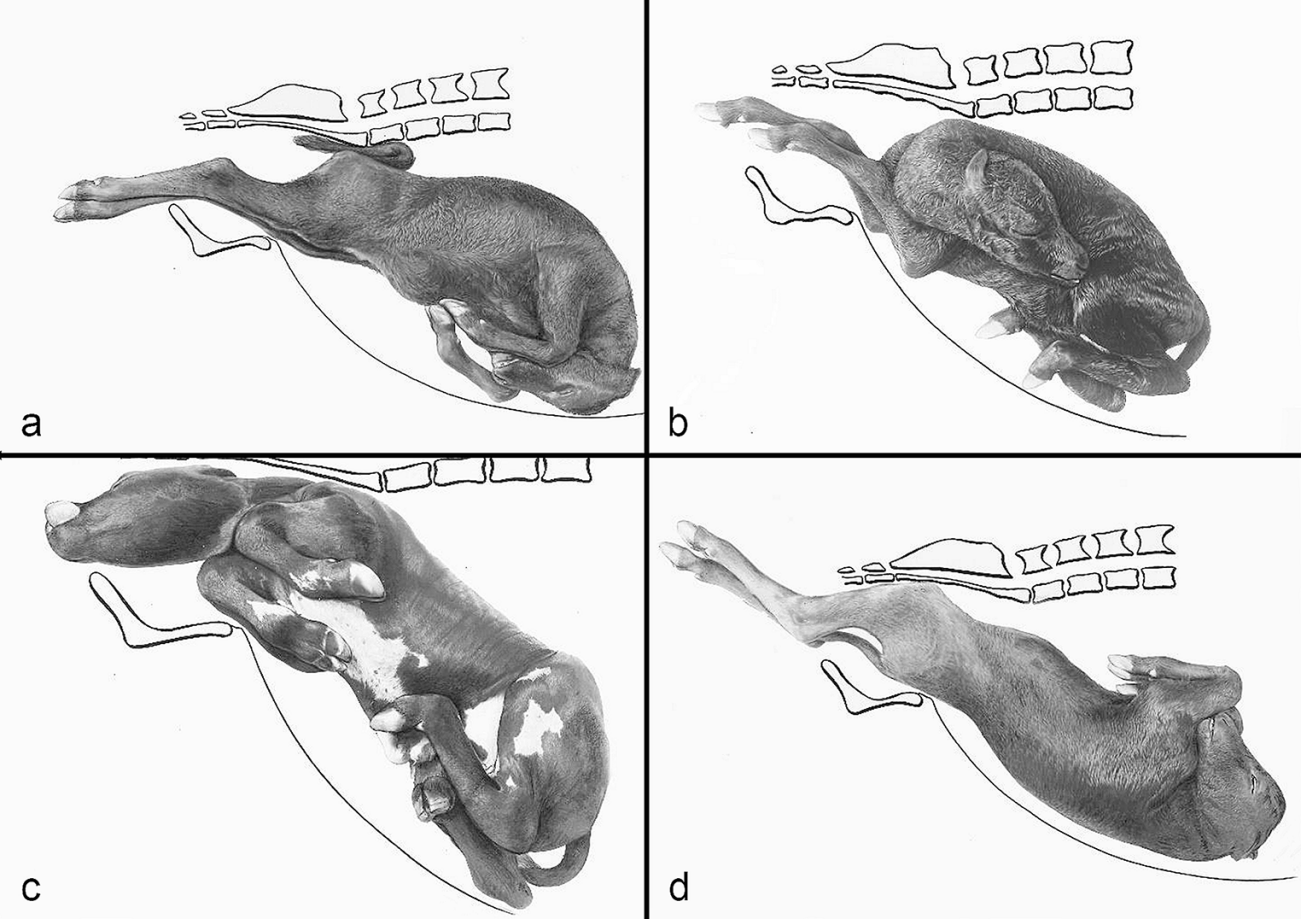
Bovine dystocia. Disposition of the calf: (**a**) Posterior presentation, dorsal position, postural defect of the tail; (**b**) Anterior presentation, dorsal position, lateral deviation of the head to the dam’s right side; (**c**) Anterior presentation, lateral position to the dam’s left side, bilateral carpal flexion; (**d**) Posterior presentation, ventral position, normal posture

**Fig. 7:**
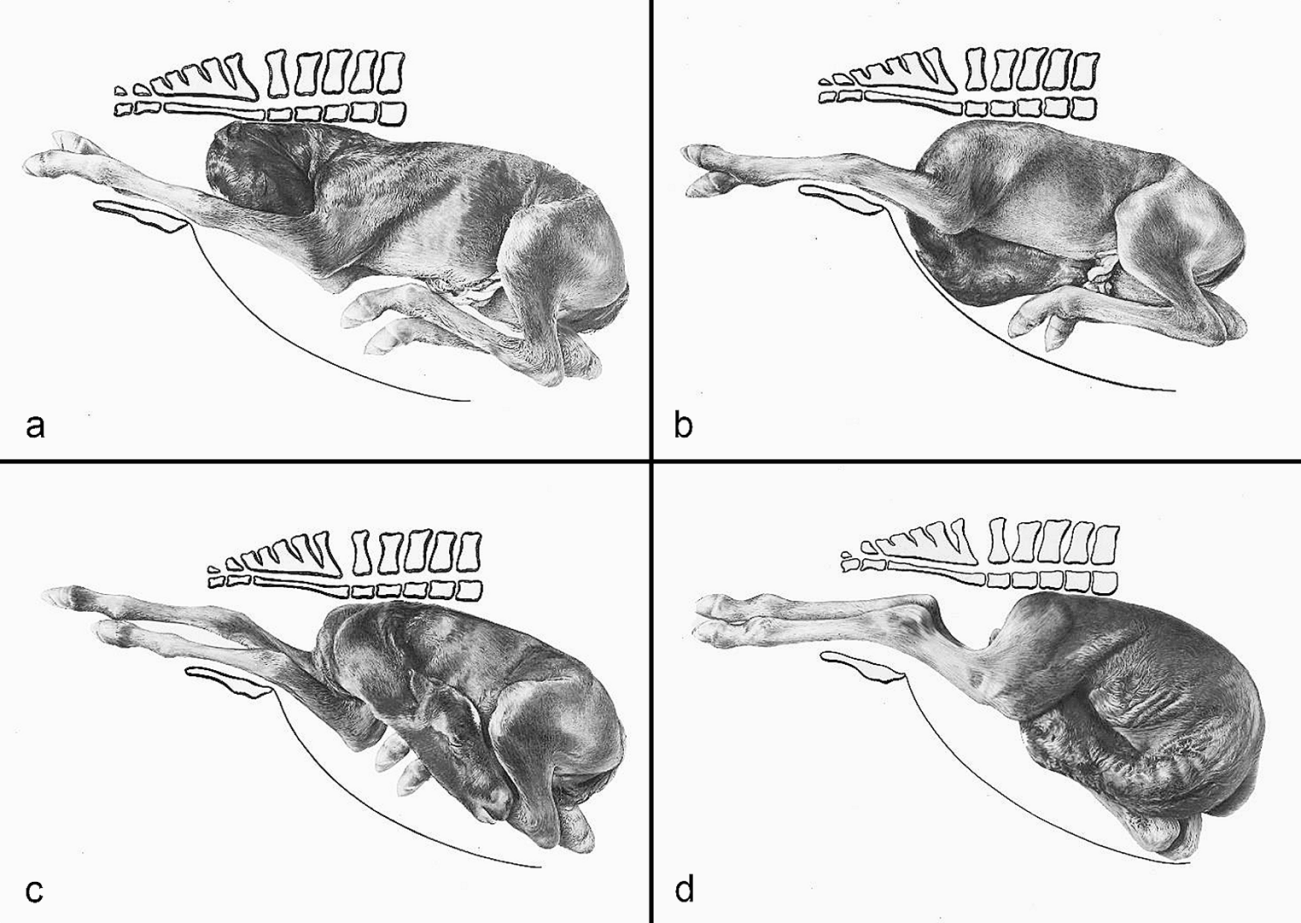
Equine dystocia. Disposition of the foal: (**a**) Anterior presentation, dorsal position, ventral deviation of the head (nape posture); (**b**) Anterior presentation, dorsal position, ventral deviation of the head (“udder posture”); (**c**) Anterior presentation, dorsal position, lateral deviation of the head to the dam’s right side; (**d**) Posterior presentation, dorsal position, ventral deviation of the head, which lies between the hindlimbs

**Fig. 8:**
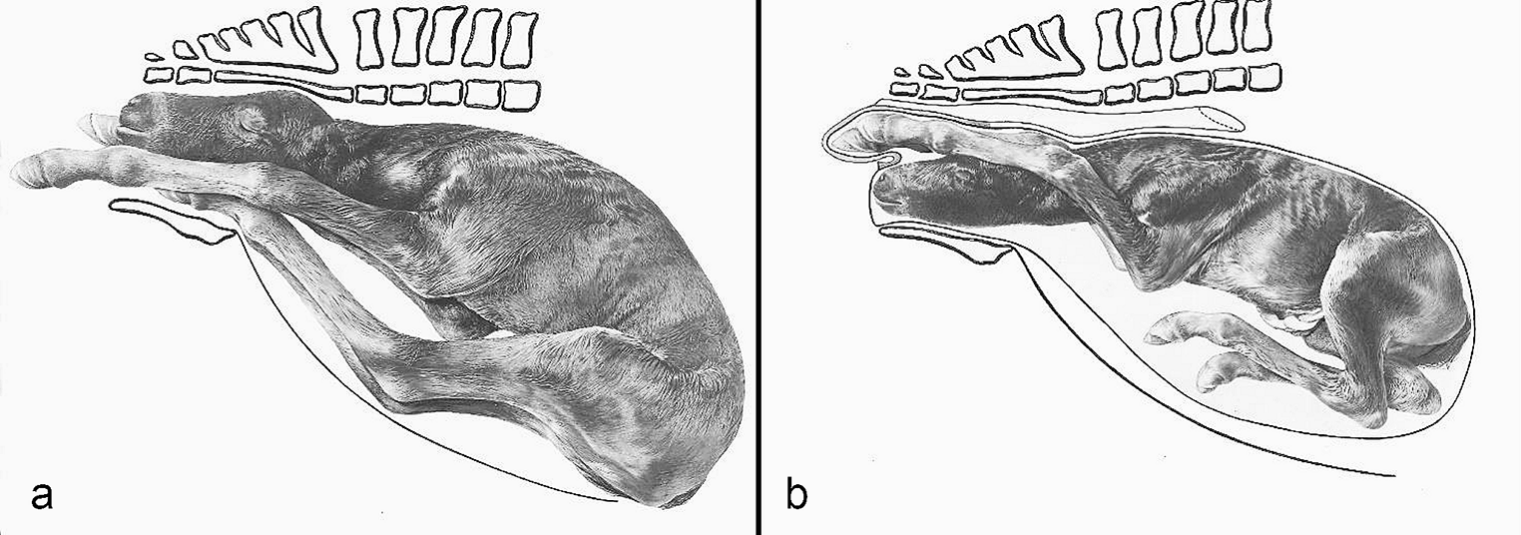
Equine dystocia. Disposition of the foal: (**a**) Ventrovertical presentation, ventrally positioned hindquarters (“Dog-sitting” position); (**b**) Anterior presentation, dorsal position, bilateral foot-nape posture

**Fig. 9:**
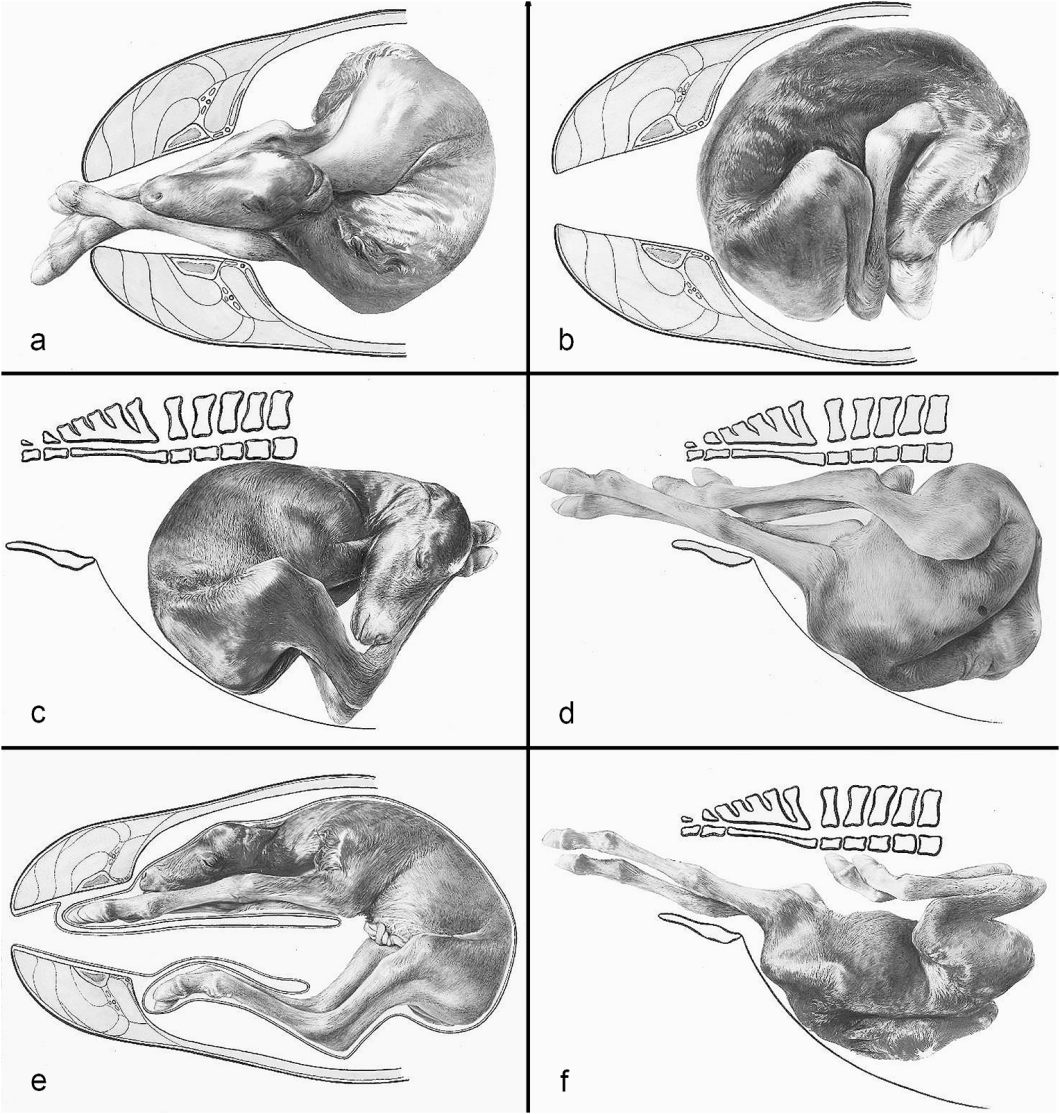
Equine dystocia. Disposition of the foal: (**a**) Ventrotransverse presentation; (**b**) Dorsotransverse presentation; (**c**) Dorsovertical presentation, ventrally positioned hindquarters; (**d**) Ventrovertical presentation, dorsally positioned hindquarters; (**e**) Ventrotransverse presentation, head and forelimbs in the left uterine horn, hindlimbs in the right uterine horn; (**f**) Anterior presentation, ventral position, lateral deviation of the head

**Fig. 10:**
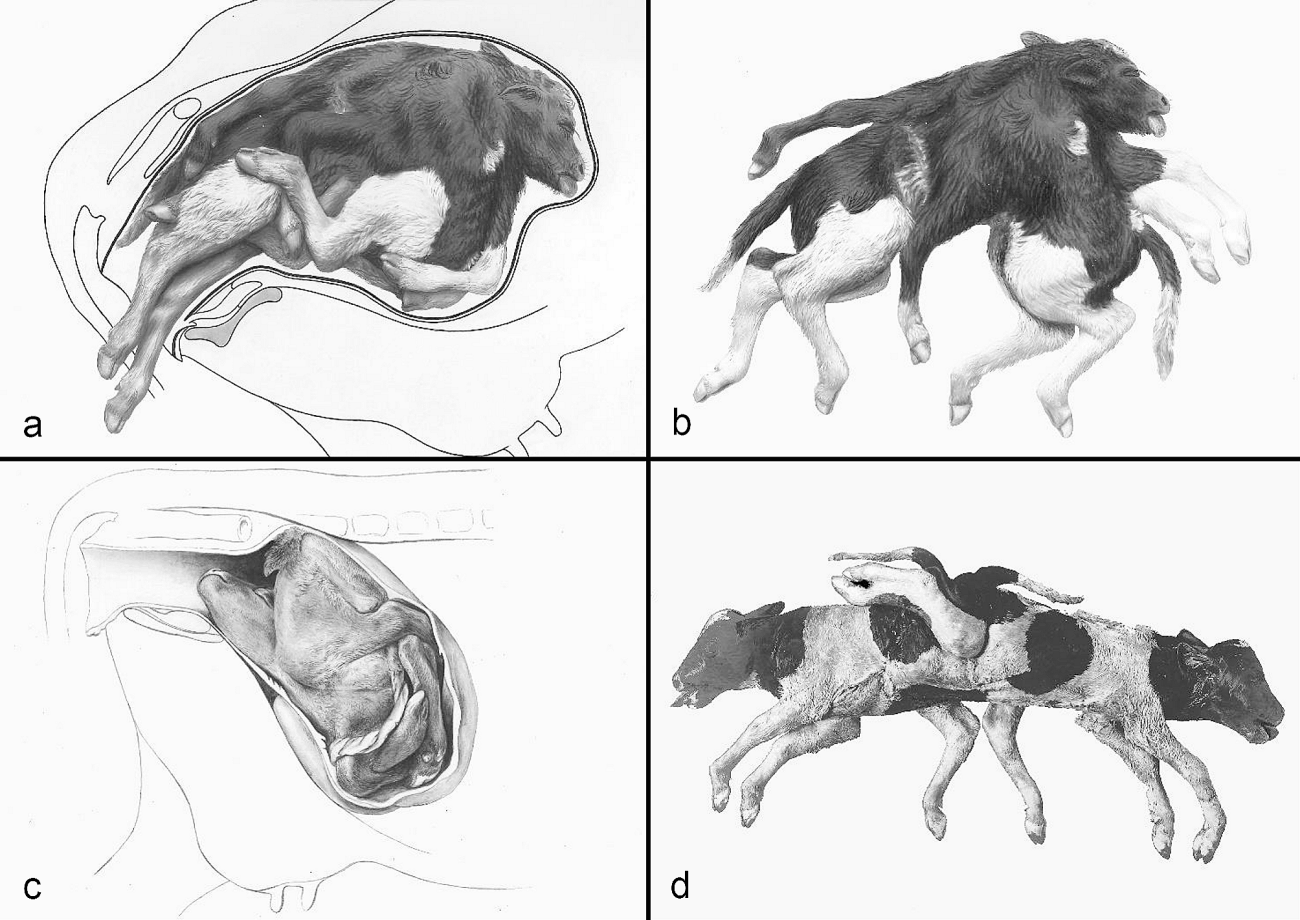
Bovine dystocia due to malformation of the fetus. (**a**) Conjoined twins (cephalothoracopagus monosymmetros) as *in utero*; (**b**) Same as (**a**) after being released from the uterus. The fetuses were extracted after euthanasia of the dam who also suffered from hydrallantois; (**c**) Contracted calf *in utero*. Part of the uterine wall and fetal membranes have been removed. The case is likely to represent a case of arthrogryposis multiplex congenita; (**d**) Ischiopagus tetrapus

The compilation of historical artworks contains seven plates showing fetal causes of dystocia in cattle and horses (Figs. 4, 5, 6, 7, 8, 9 and 10). Figures 4, 5 and 6 illustrate dystocia in cattle, while Fig. 1a displays a fetus in (normal) anterior presentation, dorsal position, normal posture. Figures 7, 8 and 9 show causes of dystocia in mares, while Fig. 10 gives examples of malformations associated with dystocia in cattle. The individual illustrations are also available in a PowerPoint presentation in Additional file 1.

### Electronic supplementary material

Below is the link to the electronic supplementary material.

**Additional file 1:** The individual illustrations of fetal causes of dystocia in cattle and horses as a PowerPoint presentation
