# Correction to: Dystocia in cattle and horses: a compilation of historical artworks dedicated to Professor Gerhard Sand (1861–1921)

**DOI:** 10.1186/s13028-024-00739-9

**Published:** 2024-04-03

**Authors:** Jørgen Steen Agerholm, Mette Christoffersen, Jan Boysen-Møller Secher, Annika Normann, Hanne Gervi Pedersen

**Affiliations:** 1 Section for Veterinary Reproduction and Obstetrics, Department of Veterinary Clinical Sciences, University of Copenhagen, Højbakkegaard Allé 5A, DK-2630 Taastrup, Denmark; 2 Department of Veterinary and Animal Sciences, University of Copenhagen, Grønnegårdsvej 15, DK-1870 Frederiksberg C, Denmark

**Acta Veterinaria Scandinavica (2024) 66:12**

10.1186/s13028-024-00733-1

Following publication of the original article [1], we have been notified that the supplementary materials’ file should be corrected as the published files were incorrectly uploaded.

The original article was updated.
